# Differential Modulation of Rhythmic Brain Activity in Healthy Adults by a T-Type Calcium Channel Blocker: An MEG Study

**DOI:** 10.3389/fnhum.2017.00024

**Published:** 2017-02-03

**Authors:** Kerry D. Walton, Emeline L. Maillet, John Garcia, Timothy Cardozo, Isaac Galatzer-Levy, Rodolfo R. Llinás

**Affiliations:** ^1^Center for Neuromagnetism, Department of Neuroscience and Physiology, New York University School of Medicine, New YorkNY, USA; ^2^Department of Biochemistry and Molecular Pharmacology, New York University School of Medicine, New YorkNY, USA; ^3^Steven and Alexandra Cohen Veterans Center for PostTraumatic Stress and Traumatic Brain Injury, Department of Psychiatry, New York University School of Medicine, New YorkNY, USA

**Keywords:** octanol, magnetoencephalography, alcohol, thalamocortical dysrhythmia, brain rhythmic activity, power spectrum

## Abstract

1-octanol is a therapeutic candidate for disorders involving the abnormal activation of the T-type calcium current since it blocks this current specifically. Such disorders include essential tremor and a group of neurological and psychiatric disorders resulting from thalamocortical dysrhythmia (TCD). For example, clinically, the observable phenotype in essential tremor is the tremor itself. The differential diagnostic of TCD is not based only on clinical signs and symptoms. Rather, TCD incorporates an electromagnetic biomarker, the presence of abnormal thalamocortical low frequency brain oscillations. The effect of 1-octanol on brain activity has not been tested. As a preliminary step to such a TCD study, we examined the short-term effects of a single dose of 1-octanol on resting brain activity in 32 healthy adults using magnetoencephalograpy. Visual inspection of baseline power spectra revealed that the subjects fell into those with strong low frequency activity (set 2, *n* = 11) and those without such activity, but dominated by an alpha peak (set 1, *n* = 22). Cross-validated linear discriminant analysis, using mean spectral density (MSD) in nine frequency bands as predictors, found overall that 82.5% of the subjects were classified as determined by visual inspection. The effect of 1-octanol on the MSD in narrow frequency bands differed between the two subject groups. In set 1 subjects the MSD increased in the 4.5-6.5Hz and 6.5–8.5 Hz bands. This was consistent with a widening of the alpha peak toward lower frequencies. In the set two subjects the MSD decrease in the 2.5–4.5 Hz and 4.5–6.5 Hz bands. This decreased power is consistent with the blocking effect of 1-octanol on T-type calcium channels. The subjects reported no adverse effects of the 1-octanol. Since stronger low frequency activity is characteristic of patients with TCD, 1-octanol and other T-type calcium channel blockers are good candidates for treatment of this group of disorders following a placebo-controlled study.

## Introduction

This study investigated the effect of a single low dose of 1-octanol (an FDA approved food-flavoring agent (FDA, 1998)) on spontaneous brain activity in healthy adults. Our interest in octanol rests on its ability to block the T-type calcium channel (Ca_v_3). This channel underlies the ability of neurons to generate low frequency oscillations and has been seen in over ten types of neurons (reviewed in (Llinás, 1988; Perez-Reyes, 2003). Indeed, this channel provides inferior olivary neurons with the intrinsic ability to oscillate at low frequencies (Llinas and Yarom, 1986). Such neuronal autorhythmicity is thought to underlie tremors such as seen in Parkinson’s disease (Sarnthein and Jeanmonod, 2007), enhanced physiological and essential tremor (Filip et al., 2016) as well as in tinnitus (De Ridder et al., 2015). In animal studies, T-type calcium channel antagonists, including 1-octanol, have been shown to reduce tremor in murine models of essential tremor (Sinton et al., 1989; Handforth et al., 2010). The T-type calcium channel also supports low frequency firing in thalamic neurons (Jahnsen and Llinas, 1984b; Cain and Snutch, 2010). Further, intracellular recordings demonstrated that 1-octanol blocks the low threshold calcium channel in central neurons (Llinás, 1988; Takahashi et al., 1989; Joksovic et al., 2007; Llinas et al., 2007; Eckle and Todorovic, 2010).

Based on the findings in animal studies, the ability of 1-octanol to reduce tremor in patients has been tested in three clinical studies. The first studies demonstrated that a single dose of 1-octanol at 1 mg/kg transiently reduced tremor in patients with essential tremor with no significant side effects or signs of intoxication (Bushara et al., 2004; Shill et al., 2004) but 1-octanol blood levels were not measured. A phase I/II clinical trial investigated the pharmacokinetics of 1-octanol at 1–64 mg/kg (*n* = 4), 64 mg/kg (*n* = 10), 128 mg/kg (*n* = 2) and found that 1-octanol was rapidly metabolized to octanoic acid (Nahab et al., 2011). Systemic levels of octanoic acid and tremor reduction peaked simultaneously, 90 min after administration (Nahab et al., 2011) and the authors suggested that octanoic acid may be an active metabolite. However, the pharmacokinetics of either 1-octanol or octanoic acid in the target organ, the brain, were not evaluated. Tremor amplitude was used as the major indicator of 1-octanol efficacies in the above studies. The aim of the present study was to find if there were changes in resting brain activity in healthy adults toward characteristics that may be viewed as harmful after taking 1-octanol. Spontaneous, rather than evoked activity was studied since abnormal brain activity is present at rest in TCD. Further, since the entire brain was of interest a study of resting activity is the best approach.

In previous studies of essential tremor 1-octanol was taken by mouth as a hard capsule (Bushara et al., 2004; Shill et al., 2004; Nahab et al., 2011) or as a soft-gel capsule (Nahab et al., 2011). A different formulation was used in this study. 1-octanol was delivered at 0.1 mg/kg by spray to the oral mucosa, much like a breath spray, allowing more controlled absorption at a significantly lower dosage.

## Materials and Methods

### Subjects

Magnetoencephalograpy (MEG) recordings were made from 32 healthy adults aged 25–74 years (19 men and 13 women; mean age 37.0 ± 2.2 years). An informed written consent was obtained from all subjects before the MEG recording and the NYU and the Bellevue Hospital Center Institutional Review Boards approved the study.

All of the subjects in this study denied neurological or psychiatric disorders, none were diabetic, and denied taking drugs that are known to influence brain activity. Due to health privacy (HIPPA) considerations and the terms of the informed consent document used, we could not explore medical reasons for any findings. One subject reported possibly falling asleep during two MEG recordings and these were excluded from the analysis.

### Study Design

Participants were recruited from the New York University Medical Center, the local community, and from a listing of participants from other studies who had agreed to be contacted. MEG recordings were made at the New York University School of Medicine Center for Neuromagnetism (CNM) located at the Bellevue Hospital Center. The subjects were weighed and the octanol dose was calculated as the number of sprays required to deliver a dose of 0.1 mg/kg. After the baseline MEG was recorded, the subjects were given the spray device and instructed to test the device by spraying into the air. They were then asked to apply into their mouth the number of sprays determined by their weight. The first subject sprayed once under the tongue so this was continued for all the subjects. They were told that the octanol would be absorbed through their mouth mucosa and asked not to swallow immediately, if possible. We did not watch the subjects to try to find when or if they made a swallowing motion. The subjects were asked to relax with their eyes close, but stay awake during each MEG recording. The subjects were monitored using a video system to ensure that they remained sitting up straight, did not move their limbs or head and did not fall asleep. Half way through the 7-min recording session they were told that this point had been reached.

### Octanol Preparation and Formulation

1-Octanol (UNII: NV1779205D) is an 8-carbon straight-chain fatty alcohol with a sharp taste and smell and naturally occurs in some plants and fruits, notably citrus. The FDA and the Council of Europe have approved 1-octanol as a flavoring agent in food and cosmetics with an accepted daily intake of 1.8 mg/kg (Class I, referenced in WHO Food Additives Series 40).

In this study the active molecule 1-octanol was formulated differently than in the previous clinical studies for essential tremors cited above. This drug was embodied for a micro-dosed oral-transmucosal delivery and was designed to overcome several limitations in comparison to prior art: (i) To be effectively micro-dosed to maximize its beneficial allosteric properties on the primary receptor target. (ii) To show tolerable organoleptic properties. (iii) To rapidly enter the blood stream and targets the brain via oral (transmucosal) delivery. Oral mucosa is a delivery route that is increasingly utilized for delivering centrally active drugs, as it evades first-pass metabolism, may increases drug bioavailability and provide opportunity for rapid drug transport to the systematic circulation (for a review see Harris and Robinson, 1992). Another advantage of intraoral absorption is that in bypassing the gastrointestinal system and being delivered at low dose, potential adverse effects of above-ADI doses of 1-octanol (Nahab et al., 2011) and/or of its metabolites to other systems or organs such has the liver and heart are prevented. (iv) To be miscible in liquid and/or solid excipients and to be stable over time. Oral transmucosal drug delivery, used since the 1980s, has been reviewed in detail by Sattar et al. (2014).

The current OMD (oral micro-dose) formulations of 1-octanol used in this study was a liquid non-pressurized “spray” medicinal form (used in this study) and formulated as 1%w/v 1-octanol therapeutic grade, 10%v/v organic vanilla beans tincture, 15%v/v Toffenut syrup, 2 mM Sucralose, 5%v/v Castor Oil, 60%v/v ethanol, completed to 100% with water. Each spray contained 1 mg of 1-octanol and was calculated to provide a transmucosal delivery of ≤0.1 mg/kg. The mean dose was 0.102 mg/kg ± 0.00086 (min. 0.094 mg/kg, max 0.109 mg/kg, variance = 0.0001, *n* = 32).

### MEG Acquisition and Analysis

Magnetoencephalograpy recordings were carried out in a mu-metal magnetically shielded room using a 275-channel instrument (CTF Systems) while the subject sat upright with their head touching the top of the recording helmet (band pass filtered 0.1–100 Hz; sample rate 600 Hz). Subjects relaxed during the two-minute break between recordings without removing their head from the recording helmet. Head position relative to the MEG sensors was measured before each recording. If this differed from that recorded before the first MEG, subjects were asked to move their head within the recording helmet to correct the discrepancy. Their posture and head position was monitored by video during each recording. Artifacts (including cardiac and eye-movements) and distant noise were reduced using a third order gradiometer (McCubbin et al., 2004). The activity of the instrument and distant noise was recorded before each session. Each 7-min recording run comprised 42 10-s trials. There were six runs for each subject: before and at 15, 30, 60, 90, and 120 min after taking 1-octanol. The subjects remained in the shielded room with the door open between recordings.

All raw data was inspected visually and movement artifacts were eliminated from further analysis by removing the corresponding 10 s trial(s). Channels were excluded from an analysis if the spectral power of the instrument and distant noise recording was ≥10fT at 2.0 Hz. Spectral analysis was performed using the Welch method. It consists of sectioning the recorded data, taking modified periodograms of these sections, and averaging these modified periodograms (Welch, 1967). Segments are overlapping (NFT, 8192; 50% overlap) and multiplied by a Hamming window function. Window functions were weighting functions applied to data to reduce the spectral leakage associated with finite observation intervals (Harris, 1978). The main advantage of this method is to reduce the variance of the spectral estimation. Head position was measured at the beginning and the end of each of the six runs for each subject. Differences in these two values greater than >0.5 cm were marked.

Before further analysis, log transformation of the power values was applied to render the distribution more normal (Gasser et al., 1982). The spectral characteristics were calculated for all sensors and for five subsets of sensors. The subsets were based on CTF sensor labeling (central, *n* = 48; frontal, *n* = 66; parietal, *n* = 42; temporal, *n* = 68; occipital, *n* = 38).

The mean alpha peak is close to 10 Hz, in the center of the alpha range of 8–12 Hz. However, the alpha peak varies across individuals over a range of 8 to 12 Hz with a standard deviation of 2.8 Hz (Haegens et al., 2014). When narrow frequency bands are analyzed, as here, the variability in the position of the alpha peak in an individual introduces an error since a range of 2.8 is greater than the width of the narrow frequency bands. Thus, to eliminate differences in the alpha peak across individuals, the individual alpha peak (IAF) (Klimesch, 1999; Klimesch et al., 1999) before the application of 1-octanol was used as the anchor point (set to 1) to establish frequency bands between 2.5 and 35 Hz for each individual. This was accomplished by first using a multipeak fit analysis to determine the IAP of each subject before 1-octanol (Igor Pro v 6.22. Multi-peak fit v2, Gauss fit, constant baseline, and range 6–14 Hz). Second, if the IAP was not 10 Hz for a subject, the power spectra for all runs for that subject were shifted along the frequency axis to center the subject’s alpha peak before 1-octanol at 10 Hz (1.0 IAP).

Five 2-Hz frequency bands: 0.25–0.45 IAF (delta), 0.45–0.65 IAF (low theta), 0.65–0.8 IAF (high theta), 0.85–1.0 IAF (low alpha), 1.0–1.25 IAF (high alpha), 1.25–1.45 IAF (beta 1), and three wider frequency bands 1.45–2.15 IAF (beta 2), 2.15–2.85 IAF (beta 3), and 2.85–3.55 IAF (beta 4) were used in determining changes after 1-octanol. An additional band, 0.95–1.15 IAP was included in discriminant analysis (described below). A lower limit of 2.5 Hz and an upper limit of 35Hz were set to eliminate instrument and far noise signals. The characteristics of the alpha peak (location, amplitude, FWHM and area) were determined using a multi-peak fit (Igor Pro v 6.22. Multi-peak fit v2, Gauss fit, constant baseline, and range 6–14 Hz). The alpha peak location in these calculations and the IAP were identical.

To determine the effect of 1-octanol inter-subject differences in absolute power were first eliminated by calculating the mean spectral density (MSD) of the log-transformed data as the total power in a frequency band/number of data points in the band. The percent change in power after 1-octanol was determined for each band as [(MSD after 1-octanol – MSD before 1-octanol)/MSD before 1-octanol] × 100.

### Statistical Analysis

Magnetoencephalograpy power spectra were compared for 32 healthy adults before (Run 1) and at five times after administration of 1-octanol (Runs 2–6) as given above. Power spectra were calculated for data from all the sensors. This 6 (runs) by 32 (subjects) data matrix was constructed to find if 1-octanol had an effect on the power spectra amplitude in 8 frequency bands and its time course.

We conducted a series of *t*-tests on the log transformed power values in the SPSS environment (SPSS Statistics, IBM) to assess the effects of 1-octanol on spectral power. First, changes in spectral power in all the MEG sensors and in five subsets of sensors after 1-octanol were calculated for run in each frequency band using the 1 sample *t*-test. Next, differences between subject groups at each time point and frequency band were calculate using the independent sample *t*-test correcting for false discovery rate with a threshold for significance after correction of at *p* ≤ 0.01 using software^1^ (Benjamini and Hochberg, 1995, 2000; Radua, 2010). Changes in power spectra were also calculated for each frequency band on the grand mean of all time points for each frequency band.

Discriminant analyses (DA) were conducted to predict whether a subject belonged to one of two groups. The classification results were cross-validated. In one DA, the predictor variables were the MSD in nine frequency bands before 1-ocanol. The grouping variable was group assignment into set 1 or set 2. This assignment was based on visual inspection of the power spectrum before 1-octanol and the alpha MSD/delta MSD ratio before 1-octanol. In another DA, the predictor variables were the change in MSD in eight frequency band at 60 min after 1-octanol. The grouping viable was the same as in the first DA. Values are given as mean ± SEM and three statistical thresholds are marked, *P* < 0.05, *P* < 0.01, *P* ≤ 0.001. (The two-tailed *t*-test was used other statistical calculations.)

## Results

### Baseline Power Spectra

The IAF for the power spectra recorded from all the sensors ranged from 8.4 to 11.2 Hz (median 10.1 Hz, mean 10.0 ± 0.013) and did not change significantly across the sensor groups or during the experiment. The IAF did not change with brain region or with 1-octanol (Supplementary Table S1).

The overall power spectra recorded from all the sensors before 1-octanol (Run 1) was visually inspected for each subject. The subjects could be divided into two broad sets, those with prominent delta and/or theta peaks and in which the alpha peak did not dominate the spectrum were assigned to set 2. Those in which the alpha peak dominated the spectrum in the absence of marked low frequency activity were assigned to set 1. These observations were expressed as the alpha MSD to delta MSD ratio.

Linear discriminant analysis was conducted to find if such a division into two sets based on visual inspection was predicted on the basis of MSD (an objective measure). The nine frequency bands were applied as the predictor variables. Significant mean differences were observed for all predictor bands except 10.5–12.5 Hz and 14.5–16.5 Hz, accounting for 61.3% of between group variability The discriminant function revealed two significant predictors; the 4.5–6.5 Hz MSD and the 2.5–4.5 Hz MSD. The cross-validated classification showed that overall 82.7% were classified correctly (i.e., as determined by the visual inspection). When the alpha to delta ratio was used as the predictor variable, cross-validated classification showed that 90.6% of the cases were correctly classified. All the cases in set 1 were correctly classified. Three cases in set 2 were predicted to be in set 1. They had alpha to delta ratios of 1.02, 1.08, 1.11. However, visual inspection revealed the presence of low frequency peaks in the power spectra for these subjects and they remained in set 2.

**FIGURE 1:**
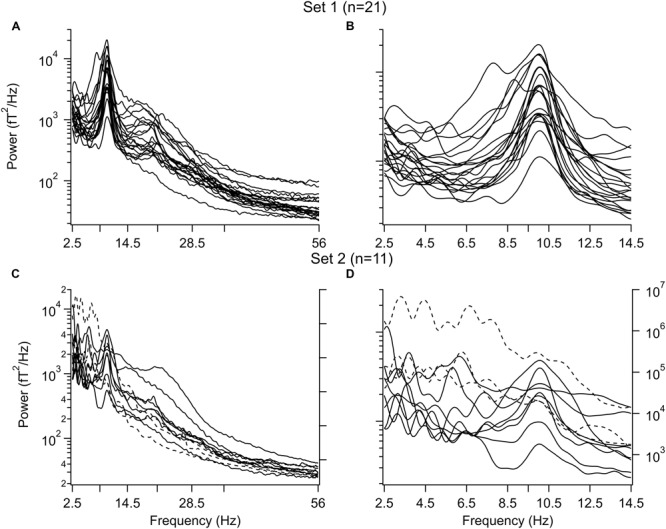
**Power spectra before 1-octanol. (A,B)** Subjects with a dominant alpha peak and without peaks in the delta and/or theta frequency band. **(A)** Entire spectrum from 2.5 to 56 Hz. **(B)** Same as in A for 2.5–14.5 Hz. **(C,D)** Subjects with peaks in the delta and/or theta frequency band. **(C)** Entire range. **(D)** Same as in C for 2.5–14.5 Hz. Note variation in waveforms. Three subjects in **(C,D)** had a larger power than the other subjects and use the power scale on the right (broken lines). Peaks were aligned to the individual alpha frequency (IAF) for each subject. This value was set to 10 Hz

The individual power spectra for all subjects are shown in **Figure 1** where the IAF was set to 10Hz and the frequency is given as the IAF x 10. Power from 2.5 to 56 Hz is shown in the left column (**Figures 1A,C**) and power from 2.5 to14.5 Hz is in the right column (**Figures 1B,D**). The difference in low frequency power between set 1 and set 2 is clear in panels B and D. (The power scale on the right in panels C and D was used for three subjects (dashed traces). Note that the power spectra for those in set 1 are quite similar while there is a great variation seen among the subjects in set 2. Due to the differences in the power spectra of the two sets further analysis was carried out separately for each set of subjects.

Considering other factors that may distinguish between the two groups. The mean age of those in set 1 was 34.9 ± 2.5 years; that for set 2 was 41.2 ± 4.3. The number of runs in which the head movement was >0.5 cm during the recording was 1.9 ± 0.33 in set 1 subjects and 2.09 ± 0.32 in set 2 subjects.

### Questions About 1-Octanol

When asked how the octanol tasted, with 1 as “very good” and 7 as “not at all good”, the mean response was 4.8 ± 0.2.

### Effect of 1-Octanol on Overall Power Spectrum

**FIGURE 2:**
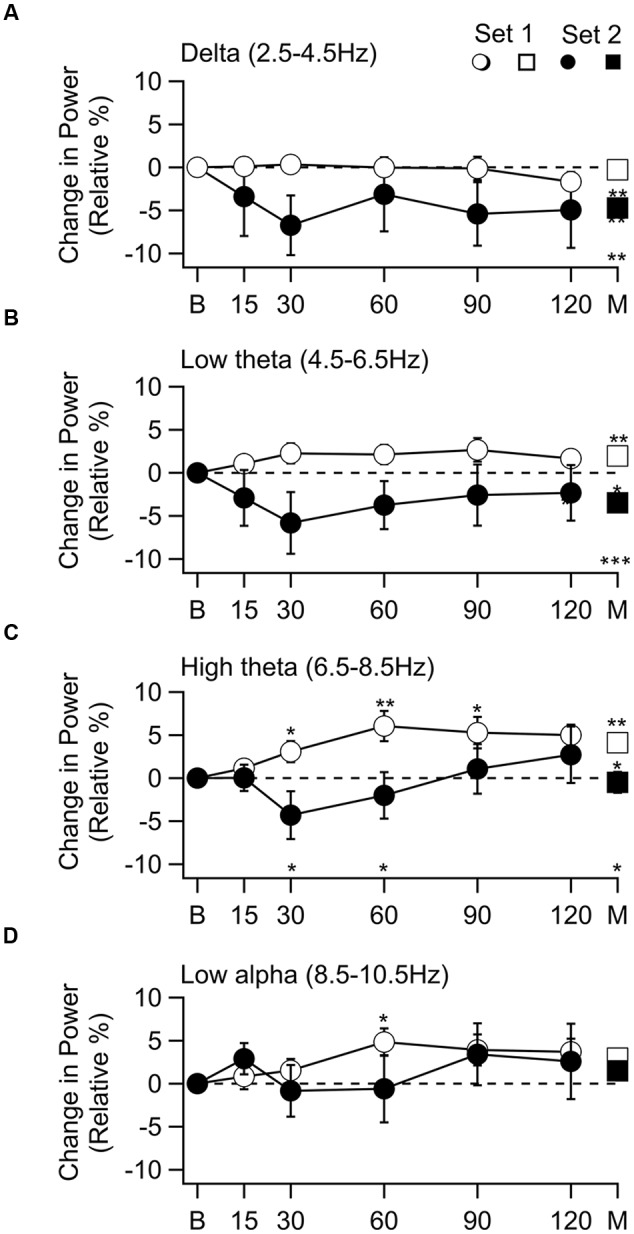
**Change in spectral power as a function of time after 1-octanol, and grand mean of all time points.** Frequency bands with a significant band are shown. **(A)** Delta band. No change in power for subjects in set 1 (○) and a decrease in power for subjects in set 2 (●). The grand mean of the mean of all time points (M), set 2 no change (□), set 2 decrease (■) difference between sets (M) was significant. **(B)** Power in low theta band. Increased in set 1 and decreased in set 2. Changes were significant in grand mean of all time points for each set (□, ■) and between sets (M). **(C)** Power in the high theta band. Increased in set 1 was significant at 30 to 120 min and in the grand mean. In set 2 power decreased at 30 and 60 min, then increased. Difference between the two sets was significant in the grand mean of all times (M). **(D**) Low alpha. Increase in set 1 was significant at 30 min. *p* < 0.05, ^*^ *p* < 0.01, ^**^ *p* ≤ 0.001 ^***^.

The percent change in mean spectral density (mean ± SEM) as a function of time after octanol delivery for subjects in set 1(○) and set 2 (●) is shown in **Figure 2** for the four frequency bands in which significant changes occurred. The grand mean change for all times is shown at the far right of each graph (M, □, ■). Significant effects were seen in the delta through low alpha ranges (**Figures 2A–D**). The effect of octanol was quite different for the subjects in set 1 and in set 2.

In the delta range, there was no effect in set 1 subjects while a decrease was seen throughout the recoding time in set 2 subjects. This decrease reached significance in the grand mean of the means for each time point (**Figure 2A**, *t*(54) = –4.12, *p* = 0.009). In the low theta range an increase was seen in set 1 subjects while there was a decrease in set 2 subjects throughout the recording period. The grand mean of these changes reached significant for set 1 (**Figure 2B** □, *t*(100) = 4.12, *p* = 0.009) and set 2 subjects [**Figure 2B** ■, *t*(54) = –4.12, *p* = 0.009]. In the high theta range the increase seen in set 1 subjects (**Figure 2C**, ○) was significant at 30 min [*t*(20) = 2.49, *p* = 0.0275, corrected], 60 min [*t*(19) = 3.43, *P* = 0.0075, corrected], 90 [*t*(20) = 2.98, *p* = 0.015, corrected] and 120 min [*t*(19) = 4.09, *p* = 0.005, corrected] as well as in the grand mean of all time points (**Figure 2C**, □) [*t*(101) = 3.43, *p* = 0.019]. Although there was a decrease followed by an increase in the set 2 subjects, these changes did not reach significance. In the low alpha range the only significant change was an increase in the set 1 subjects 60 min after 1-octanol administration [**Figure 2D**, ○, *t*(19) = 3.03, *p* = 0.035, corrected].

When the two sets were compared (^∗^ above abscissa), there was a difference in the high theta range (**Figure 2C**) at 30 min *t*(30) = 2.81, *p* = 0.04, corrected) and 60 min [*t*(28) = 2.57, *p* = 0.04, corrected]. The difference between the grand means of the two sets was significant in the delta [*t*(154) = 3.7, *p* = 0.004], low theta [*t*(153) = 5.19, *p* = 0.0001], and high theta [*t*(154) = 2.72, *p* = 0.022] ranges.

Significant increases were seen in the 6.5–8.5 Hz [*t*(154) = 3.20, *p* = 0.002) and 8.5–10.5 Hz [*t*(154) = 3.9.4, *p* = 0.0001] when all the subjects were analyzed together. This is consistent with the observation of increased power in both set 1 and 2 subjects at 90 and 120 min after 1-octanol in these frequency ranges (**Figures 2C,D**).

**FIGURE 3:**
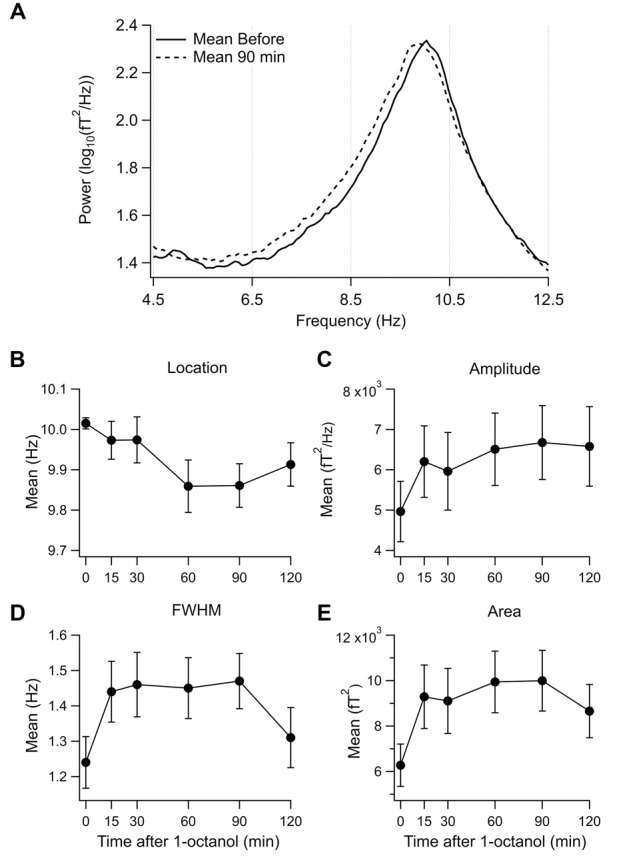
**Effect of 1-octanol on the alpha peak for set 1 subjects. (A)** Superposition of mean power spectrum before (solid line) and 90 min (broken line) after 1-octanol for set 1 subjects. Note broadening of alpha peak to lower frequencies. **(B–E)** Plots of characteristics of the alpha peak as a function of time after 1-octanol. None reached significance but they show time course and trends following 1-octanol. FWHM, full width at half maximum.

To visualize the changes in the power spectrum in the set 1 subjects after 1-octanol, the mean power spectra were compared before (solid line) and at 90 min (broken line) after application of 1-octanol (**Figure 3A**). Note that there is a widening of the alpha peak toward lower frequencies. For set 1 subjects, analysis was carried out for the mean location (**Figure 3B**), amplitude (**Figure 3C**), full width at half maximum (FWHM, **Figure 3D**) and area under a curve fitted to the alpha peak (**Figure 3E**). When corrected for false discovery rate, none of the changes shown in **Figures 3B–E** reached significance. However, these plots do show clearly the trend and time course of the changes in the characteristics of the alpha peak after 1-octanol. A similar analysis of data from subjects in set 2 was not meaningful due to the great variability in the power spectra (See **Figures 1C,D**).

A discriminant analysis was conducted to find if the effect of 1-octanol on power spectra could be used as a predicted of a subject’s membership in set 1 or set 2. The predictor variables were changes in MSD in nine frequency bands (between 2.5 and 35 Hz). Based on the time course of the 1-octanol effect (**Figure 2**), values at 60 min after administration of 1-octanol were used. Significant mean differences were observed for all predictor bands between 2.5 and 10 Hz, accounting for 64.8% of between group variability. The cross-validated classification showed that overall 78.8% subjects were correctly classified.

### Distribution of Changes in Power Spectra

To obtain an estimate of the distribution of changes in spectral power among cortical regions following 1-octanol, the percent change in mean spectral density was calculated for five sets of sensors (grand mean of the means for each time point for sensors on the right and those on the left side, *n* = 10). **Table 1** includes only those changes in power that reached significance at the *p* ≤ 0.01 level (correct for FDR). All significant changes in set 1 subjects were increases while those in set 2 subjects were decreases. Most changes for both sets of subjects were in the parietal and occipital sensors.

**Table 1:** Summary of significant changes in spectral power after 1-Octanol in five sets of MEG sensor groups for set 1 and set 2 subjects and comparison of sets.

		Sensor Set								

**Band**	**Set**	**Front**		**Center**	**Parietal**		**Temporal**		**Occipital**	
Delta	1	–	^∗^	–	1.1 ± 0.17	^∗^	–	^∗^	1.4 ± 0.23	^∗^
	2	–3.5 ± 0.54		–	–3.0 ± 0.72		–4.8 ± 0.46		–4.9 ± 0.56
Low Theta	1	–	^∗^	–	1.4 ± 0.18	^∗^	0.59 ± 0.12	^∗^	2.3 ± 0.22	^∗^
	2	–1.6 ± 0.42		–	–		–3.3 ± 0.32		–3.2 ± 0.41	
High Theta	1	0.83 ± 0.25		1.5 ± 0.33	2.5 ± 0.32	^∗^	2.0 ± 0.30	^∗^	3.6 ± 0.31	^∗^
	2	–		–	–		–		–	
Low Alpha	1	1.5 ± 0.26		1.8 ± 0.28	1.8 ± 0.24	^∗^	2.0 ± 0.20	^∗^	1.8 ± 0.21	^∗^
	2	–		–	–		–		–2.0 ± 0.33	
High Alpha	1	1.0 ± 0.11		0.70 ± 0.18	–	^∗^	0.64 ± 0.15	^∗^	–	^∗^
	2	–		–	–1.3 ± 0.40		–		–2.6 ± 0.32	
Beta 1	1	–		–	–	^∗^	–		–	^∗^
	2	–		–	-1.9 ± 0.46		–		–2.3 ± 0.13	
Beta 2	1	–		–	0.85 ± 0.14	^∗^	0.70 ± 0.13		1.4 ± 0.17	^∗^
	2	–		–	–2.0 ± 0.50		–		–2.0 ± 0.14	
Beta 3	1	–		–	–	^∗^	–		0.98 ± 0.17	^∗^
	2	–		–	–1.9 ± 0.46		–		–1.5 ± 0.21	
Beta 4	1	–		–	–	^∗^	–		1.2 ± 0.19	^∗^
	2	–		–	–2.8 ± 0.50		–		–	

*All values *p* ≤ 0.01, significant difference between set 1 and set 2 subjects are marked by an ^∗^.*

The largest power increases in set 1 subjects were in the high theta band (4-6Hz). There was an increase in every sensor set in the high theta and low alpha bands (6–8 Hz, 8–10 Hz). In set 2 subjects, the largest decrease was in the delta band (2–4 Hz). Unlike the set 1 subjects, decreases were also seen in the higher frequency ranges.

Head movement did not contribute to the distribution of activity after octanol since the occurrence of movement >0.5 cm did not change significantly with time during the study (run number) in either set of subjects. Further, such an effect would be expected to be seen (as an increase or decrease) across all frequency bands, which was not the case.

## Discussion

### Baseline Power Spectrum

The present results show that the effect of 1-octanol on the brain activity of healthy adults depends on the characteristics of their baseline power spectra. The brain baseline power spectrum, dominated by the alpha rhythm in 21 subjects, was localized to posterior brain regions expected in healthy adults (Nunez et al., 2001; Basar, 2012). Alpha oscillations are proposed to originate in the lateral thalamus and maintained through a thalamocortical-thalamus loop that includes the reticular thalamus (Steriade, 2000, 2001; Schreckenberger et al., 2004). This may represent one of several phenomena in the alpha band, with separate origins and localizations (see (Feshchenko et al., 2001; Basar, 2012).

Unexpectedly, strong delta (<4 Hz) and theta (4–8 Hz) rhythms were seen in 11 subjects. Low frequency activity was larger in amplitude than that in the alpha band in 5 of these subjects, one subject did not have a peak in the alpha range. Such low frequency oscillations also have a thalamic origin and are generated when T-type calcium channels are de-inactivated by membrane hyperpolarization (Jahnsen and Llinas, 1984a,b; McCormick and Pape, 1990; McCormick and Huguenard, 1992). In addition to the thalamus (Jones, 2007), the hippocampus is a well-known source of theta oscillations (Kahana et al., 2001; Anderson, 2010).

Although low frequency thalamic activity is seen in awake adults, and may play a role in stimulus detection see (Sherman, 2005), it is not expected under the conditions of these recordings. Rather, delta rhythms (0-4Hz) are most commonly associated with stage 3-4 sleep and theta rhythms are associated with sleep and drowsiness (McCormick and Bal, 1997; Knyazev, 2012).

Low frequency oscillations are generally seen in neurological and psychiatric patients (Knyazev, 2012) and can be considered, given their dynamics, characteristics of thalamocortical dysrhythmia (TCD) (Llinas et al., 1999; Jeanmonod et al., 2001). Persistent thalamic neuronal hyperpolarization leading to TCD following de-inactivation of T-type calcium channels, may occur by excess inhibition (Llinas and Jahnsen, 1982), or by disfacilitation resulting from thalamic deafferentation (Llinas et al., 1998; Wang and Thompson, 2008). It may also occur following block of excitatory ligand-gated channels (Zhang et al., 2009).

In this study, low frequency power was largest in the temporal sensors in 79 % of the subjects. Temporal low voltage irregular delta waves (TLID) are found in early stages of cardiovascular damage (Inui et al., 2001) and in the left temporal lobe of diabetics (Inui et al., 1998). They are also commonly found in the elderly and their incidence increases with age (Motomura et al., 2008). Spontaneous low frequency oscillations in patients in pain (Schulman et al., 2005; Walton et al., 2010) and with psychiatric disorders, including depression, were localized to mesial frontal regions as well as somatotopically appropriate cortex (Schulman et al., 2011). Hippocampal theta oscillations have also been reported in depressed patients (Cornwell et al., 2010).

The ionic mechanisms and anatomical origin for the oscillations seen in these subjects, while probably related to the activation of low-threshold calcium channels (Llinas et al., 1999), remains unexplained. Further studies that incorporate MRI and a clinical evaluation could be contemplated in healthy controls to determine the origin of the low frequency oscillations seen in this study.

### Effect of 1-Octanol on Subjects with an Alpha Dominated Power Spectrum

Although both electroencephalographic (EEG) and MEG studies have reported that ethanol modifies spontaneous brain activity in several frequency bands, the results are inconsistent. Indeed, a meta-analysis of studies on the acute effect of alcohol in healthy adults found increased, decreased, or no change in all the frequency bands studied (Zoethout et al., 2011). This inconsistency is often seen in EEG and MEG studies and may be due, primarily, to a lack of standardization in the definition of the frequency bands, the selection of cortical areas, or in the electrode position.

The most marked action of 1-octanol administration in set 1 subjects was the increase in spectral power in the high theta band. Indeed, an increase in 6.5–8.5 Hz activity was recorded across all sensor groups. When sets of particular sensor groups were considered, rather than all the sensors, an increase was seen in the low alpha range as well. By contrast, a reduction in alpha frequency has been reported to accompany increased alpha amplitude with ethanol (Ehlers et al., 1989; Stenberg et al., 1994; Klimesch, 1999; Kahkonen, 2005; Nikulin et al., 2005; Stoffers et al., 2008; Bosboom et al., 2009; Zoethout et al., 2011). While the above shift in the alpha peak was not seen here, such findings are consistent with the broadening of the alpha peak and the increased amplitude in the high theta band. Differences from ethanol may also be due, in part, to the use of narrow frequency bands that revealed changes after 1-octanol that were obscured when an alpha band of 8–13 Hz was used.

In addition to the above, an increase in the theta band power has been reported after ethanol (Lukas et al., 1986; Ehlers et al., 1989; Stenberg et al., 1994). Since 1-octanol is known to block T-type calcium channels (Llinás, 1988), one must consider that the increased low frequency oscillations seen in this group of subjects may have another origin.

1-Octanol increased spectral power in the high theta and low alpha bands (6–10 Hz) across the entire MEG sensor sets and was highest in the occipital group (**Table 1**). This is in general agreement with a MEG study of the effect of ethanol on resting activity that reported an increased activity in spectral power in the alpha band (8–10 Hz) in occipital cortex (Nikulin et al., 2005).

The changes in spectral power were first noted at 30 min and peaked at 60 min after administration. The effect of 1-octanol to reduce tremor peaked at 90 min after ingestion (Nahab et al., 2011). The earlier peak effect seen in this study is consistent with the method of delivery that would be expected to be faster than ingestion.

### Effect of 1-Octanol on Subjects With Low Frequency Activity

The ability of 1-octanol to directly reduce low frequency firing and thus spectral power was seen in the group of subjects in whom low frequency activity predominated (set 2). Indeed, rather than no change or an increase as seen in set 1 subjects, a decrease was seen throughout the recording period in the lowest frequency bands (2.5–6.5 Hz). This is in contrast to the changes in the higher frequency bands (**Figures 2C,D**) where the initial decrease in MSD was followed by an increase.

From an electrophysiological perspective, the ability of 1-octanol to decrease low frequency power is consistent with its ability to block the Cav3.1 T-type calcium channels in thalamocortical neurons (Eckle and Todorovic, 2010) and Cav3.3 T-type calcium channels in thalamic reticular neurons (Joksovic et al., 2010). These results are also consistent with the ability of this alcohol to reduce synchronization of neocortical networks (Bostanci and Bagirici, 2006; Gigout et al., 2006) and to block gap junctions (Pappas et al., 1996). (However, the effects on gap junctions require a higher dosage that required for Cav3.1 channel block.)

The effect of 1-octanol to reduce oscillations in the 2.5 to 6.5 Hz range was seen in the frontal, temporal, and occipital sensor groups. Consideration of the neuronal basis for the localization of changes in spectral power and the difference between set 1 and set 2 in this regard would be highly speculative. Sensor groups give an approximation of source activity, independent component, or a similar analysis is needed to localize magnetic sources in the brain.

The decrease in power at higher frequencies seen in the sensor groups has also been observed with ethanol (Nikulin et al., 2005), but an increase (Stenberg et al., 1994) has also been reported. This may be related to the finding that the effect of ethanol on T-type calcium channels is dose dependent (Mu et al., 2003).

### Limitations and Outlook

This was a proof of concept study, to find if 1-octanol when delivered at a low dose, using an oral transmucosal non-pressurized spray was harmful or changed oscillatory brain activity in healthy adults. All the subjects received the same dose of 1-octanol, normalized by body weight. Thus, limitations of the study are; (a) there was not a placebo control, (b) only one dose was used, (c) the absorbed dose was not measured and may have varied across subjects, and (d) pharmokinetics were not included in the study design. Thus, although it can be suggested that the effects were due to the octanol, this is not certain. Indeed, if the changes are due to the passage of time with accompanied change in attention, or expectation, cannot be ruled out. An unplanned control was the inclusion of subjects with abnormal low frequency activity (set 2) and the differential effect of 1-octanol on the two groups of subjects. Importantly, this introduces a potential limitation to the interpretation of the current results. Specifically, the differential rate of change between the two groups could be a function of regression to the mean as the group with the most significant change also demonstrated the highest baseline scores on low frequencies. However, we cannot adjudicate this question based on the data at hand. This limitation reflects the pilot nature of this studied where only healthy individuals are being examined. Future studies will address this limitation by utilizing a larger, placebo controlled, double blind study that includes source location is needed to confirm the effects seen in this study and identify the sources of such changes in brain oscillations.

## Concluding Remarks

The goal of the study was to find if 1-octanol, given in the dose and method applied, would lead to changes in the power spectra and behavioral measures of healthy adults toward characteristics that may be viewed as harmful. The subjects reported no harmful effects and 1-octanol did lead to changes in spectral properties in set 1 subjects that were largely consistent with what has been seen after administration of ethanol (Zoethout et al., 2011). The unexpected inclusion of a small number of subjects (*n* = 11) with low frequency spectral activity provided an opportunity to suggest that 1-octanol did indeed block such activity. Thus, 1-octanol or other T-type calcium channel blockers are promising candidates for further studies of disorders involving the abnormal activation of the T-type calcium current as reviewed in (Iftinca, 2011).

We have taken a new approach to the delivery of the active component of 1-octanol as a micro-dosed formulated drug and of its prospect to aid and/or mitigate a number of medical conditions. Dietary supplements, OTC medicines, and phytotherapeutic drugs are commonly used and valued for their functional and medicinal properties. The usage of the natural substance 1-octanol embodied in an efficacious low-dosage formula holds promises for development as a plant-derived medicine.

## Ethics Statement

This study was carried out in accordance with the recommendations of the NYU and the Bellevue Hospital Center Institutional Review Boards. All subjects read and signed an informed written consent in accordance with the Declaration of Helsinki. No vulnerable populations participated in this study.

## Author Contributions

RL, KW, EM, and TC designed the study. EM formulated and prepared the 1-octanol. KW conducted the study. JG wrote and ran the programs used for data analysis. KW and RL drafted the manuscript and figures. IG-L carried out the statistical analysis. All authors contributed to editing and approved the manuscript.

## Conflict of Interest Statement

The authors declare that the research was conducted in the absence of any commercial or financial relationships that could be construed as a potential conflict of interest.

## Footnotes

1 http://www.sdmproject.com
