# Supplementary material for: Differential Modulation of Rhythmic Brain Activity in Healthy Adults by a T-Type Calcium Channel Blocker: An MEG Study
Source: Front Hum Neurosci. 2017 Feb 3;11:24. doi: 10.3389/fnhum.2017.00024 (PMC5289965; doi:10.3389/fnhum.2017.00024)
Supplement: Supplementary file 1 [file Table_1.DOCX]

Supplementary Table 1. Individual alpha peak values (Hz) for sensor groups before octanol.

Baseline before 1-octanol

| Sensors | Minimum | Maximum | Median | Range | n |
| --- | --- | --- | --- | --- | --- |
| All | 8.4 | 11.2 | 10.1 | 2.8 | 32 |
| Central | 8.7 | 11.4 | 10.3 | 2.6 | 31 |
| Frontal | 8.4 | 11.1 | 10.2 | 2.7 | 31 |
| Occipital | 8.5 | 11.2 | 10.1 | 2.7 | 32 |
| Parietal | 8.7 | 11.3 | 10.1 | 2.6 | 32 |
| Temporal | 8.1 | 11.2 | 10.1 | 3.1 | 32 |

15 min after 1-octanol

| Sensors | Minimum | Maximum | Median | Range | n |
| --- | --- | --- | --- | --- | --- |
| All | 8.4 | 11.1 | 10.1 | 2.7 | 32 |
| Central | 8.5 | 11.0 | 10.3 | 2.4 | 28 |
| Frontal | 8.2 | 11.0 | 10.1 | 2.8 | 29 |
| Occipital | 8.3 | 11.1 | 10.1 | 2.8 | 31 |
| Parietal | 8.5 | 11.3 | 10.2 | 2.8 | 31 |
| Temporal | 8.7 | 11.0 | 10.1 | 2.4 | 32 |

30 min after 1-octanol

| Sensors | Minimum | Maximum | Median | Range | n |
| --- | --- | --- | --- | --- | --- |
| All | 8.3 | 11.1 | 10.0 | 2.7 | 32 |
| Central | 8.8 | 11.7 | 10.2 | 2.9 | 30 |
| Frontal | 8.0 | 11.6 | 10.1 | 3.6 | 31 |
| Occipital | 8.3 | 11.0 | 10.1 | 2.7 | 32 |
| Parietal | 8.5 | 11.3 | 10.1 | 2.7 | 31 |
| Temporal | 8.2 | 11.0 | 10.0 | 2.9 | 32 |

60 min after 1-octanol

| Sensors | Minimum | Maximum | Median | Range | n |
| --- | --- | --- | --- | --- | --- |
| All | 8.3 | 11.1 | 10.0 | 2.7 | 32 |
| Central | 8.6 | 11.2 | 10.0 | 2.7 | 31 |
| Frontal | 8.4 | 10.9 | 9.9 | 2.5 | 30 |
| Occipital | 8.2 | 11.0 | 10.0 | 2.8 | 31 |
| Parietal | 8.5 | 11.2 | 10.0 | 2.7 | 31 |
| Temporal | 8.3 | 11.0 | 9.9 | 2.6 | 32 |

90 min after 1-octanol

| Sensors | Minimum | Maximum | Median | Range | n |
| --- | --- | --- | --- | --- | --- |
| All | 8.2 | 10.9 | 10.0 | 2.7 | 32 |
| Central | 8.0 | 11.2 | 10.2 | 3.1 | 31 |
| Frontal | 7.9 | 10.8 | 10.1 | 2.9 | 31 |
| Occipital | 8.2 | 10.9 | 10.0 | 2.7 | 32 |
| Parietal | 8.4 | 11.0 | 10.1 | 2.7 | 32 |
| Temporal | 8.1 | 10.8 | 9.92 | 2.7 | 31 |

120 min after 1-octanol

| Sensors | Minimum | Maximum | Median | Range | n |
| --- | --- | --- | --- | --- | --- |
| All | 8.3 | 11.2 | 10.0 | 2.9 | 31 |
| Central | 8.2 | 11.5 | 10.2 | 3.3 | 30 |
| Frontal | 8.4 | 11.1 | 10.0 | 2.8 | 31 |
| Occipital | 8.1 | 11.2 | 10.1 | 3.1 | 32 |
| Parietal | 8.3 | 11.3 | 10.0 | 3.0 | 32 |
| Temporal | 8.3 | 11.1 | 10.0 | 2.8 | 31 |
